# Maternal anemia is a potential risk factor for anemia in children aged 6–59 months in Southern Africa: a multilevel analysis

**DOI:** 10.1186/s12889-018-5568-5

**Published:** 2018-05-22

**Authors:** Peter A. M. Ntenda, Owen Nkoka, Paul Bass, Thomas Senghore

**Affiliations:** 1 School of Public Health, College of Public Health, Taipei Medical University, No.250, Wu-Hsing St, Taipei City, 110 Taiwan, R.O.C.; 2 School of Medicine and Allied Health Sciences, University of The Gambia, P.O. Box 1646, Independence Drive, Banjul, The Gambia

**Keywords:** Malawi, Mozambique, Namibia, Zimbabwe, Maternal anemia, Childhood anemia, Multilevel

## Abstract

**Background:**

The effect of maternal anemia on childhood hemoglobin status has received little attention. Thus, we examined the potential association between maternal anemia and childhood anemia (aged 6–59 months) from selected Southern Africa countries.

**Methods:**

A cross-sectional study using nationally representative samples of children aged 6–59 months from the 2010 Malawi, 2011 Mozambique, 2013 Namibia, and 2010–11 Zimbabwe demographic and health surveys (DHS) was conducted. Generalized linear mixed models (GLMMs) were constructed to test the associations between maternal anemia and childhood anemia, controlling for individual and community sociodemographic covariates.

**Results:**

The GLMMs showed that anemic mothers had increased odds of having an anemic child in all four countries; adjusted odds ratio (aOR = 1.69 and 95% confidence interval [CI]:1.37–2.13) in Malawi, (aOR = 1.71; 95% CI: 1.37–2.13) in Mozambique, (aOR = 1.55; 95% CI: 1.08–2.22) in Namibia, and (aOR = 1.52; 95% CI: 1.25–1.84) in Zimbabwe. Furthermore, the odds of having an anemic child was higher in communities with a low percentage of anemic mothers (aOR = 1.52; 95% CI: 1.19–1.94) in Mozambique.

**Conclusions:**

Despite the long-standing efforts to combat childhood anemia, the burden of this condition is still rampant and remains a significant problem in Southern Africa. Thus, public health strategies aimed at reducing childhood anemia should focus more on addressing infections, and micronutrient deficiencies both at individual and community levels in Southern Africa.

**Electronic supplementary material:**

The online version of this article (10.1186/s12889-018-5568-5) contains supplementary material, which is available to authorized users.

## Background

Anemia, defined as the volume of packed red blood cells/hematocrit (Hct) or hemoglobin (Hb) concentration greater than two standard deviation (2SD) below mean for age, may be due to three general causes (i.e. blood loss, increased red blood cell (RBC) destruction or reduced RBC production) [1, 2]. The major physiologic impact of anemia is reduced oxygen delivery to tissues, resulting in both compensatory responses and acute or chronic consequences including poor growth, decreased activity, impaired cognitive performance, behavioral, motor development and limited cardiovascular reserve [1, 3–5].

Anemia is a major public health problem in developing countries and a direct cause of childhood mortality and morbidity [6, 7]. Globally, 43% of preschool-age children are anemic [5, 8, 9] and 28.5% of these children reside in sub-Saharan Africa (SSA), which presents a startling prevalence rate of 67% [10]. The causes of anemia are multifactorial, however, about 50% of all anemia cases are due to iron deficiency [11, 12], although other factors such as micronutrient deficiencies (i.e., folate, riboflavin, and vitamins A and B12) [12–14], acute and chronic inflammation (i.e., malaria, tuberculosis, and HIV) [13, 15, 16], and inherited or acquired disorders that affect Hb synthesis, RBC production, or RBC survival (i.e., hemoglobinopathies) [15, 17], can all be etiologies of anemia. In addition to nutritional and pathological factors, previous researchers have demonstrated other factors such as a child’s characteristics [11, 18–20], maternal characteristics [18–20], household characteristics [19, 20], and community characteristics [20], also have impacts on childhood anemia.

The World Health Organization (WHO) estimated that over 50% of all women living in developing countries are anemic, compared with 18% in industrialized countries [21]. However, the burden of this condition is more pervasive in Asia and Africa where 60 and 52% of women are estimated to be anemic respectively [21]. Previous studies have reported that maternal anemia during pregnancy is associated with a higher risk of low birth weight, preterm birth, perinatal and neonatal mortality, maternal morbidity and mortality, and low productivity [22–24]. Regardless, a large body of research has established links between maternal anemia in pregnancy and poor infant birth outcomes. However, little is known whether maternal anemia, in general, has an influence on childhood anemia after delivery. Thus, we aimed to investigate whether there is an association between maternal anemia and childhood anemia in four Southern African countries, controlling for a wide range of individual- and community-level sociodemographic factors.

## Methods

### Study design, sampling technique, and data collection

This study utilized demographic and health survey (DHS) data from four Southern African countries (2010 Malawi, 2011 Mozambique, 2013 Namibia, and Zimbabwe 2010–11). Methodologies used in these surveys have been previously described [25]. In brief, the surveys utilized a stratified two-stage cluster design. In the first stage, clusters were randomly selected from master sampling frames (enumeration area). The second stage selected a systematic sample of households from the communities (clusters). Information was collected from women aged 15–49 years, who had under-5 children prior to the survey. With the aid of an interviewer administered questionnaire information on sociodemographic, economic, environmental, immunization, anthropometric, household characteristics, child health care and population health indicators were collected. All the above surveys had response rates of more than 90%. A random procedure was conducted to select one child per mother to avoid the clustering effects, which generated final sample sizes of 2507, 1933, 1116, and 2578 in Malawi, Mozambique, Namibia and Zimbabwe respectively. The HemoCue blood hemoglobin system (HemoCue 201+; Ängelholm, Sweden) [26] was utilized for Hb testing for children and mothers using finger prick blood. The HemoCue system is a rapid and widely used system in both clinical and survey settings that is comparable to standard laboratory techniques [27].

### Measures

#### Outcome variable

Anemia in children under the age of 5 was the outcome variables in this study, defined as per WHO recommendations (Hb < 11 g/dL) [1, 3].

#### Main independent variables

The main predictor variable of this study was maternal anemia, used as both continuous (Hb concentration) and categorical variable (cut-off of < 12 g/dL as per WHO [1, 3]).

### Covariates

#### Individual-level/maternal and household-level factors

We selected covariates to adjust for possible confounding factors in the analyses (control variables). There were a total of twelve individual/household-level variables included in this study. Child-specific factors included child’s sex (male or female), child’s age (in months) (6–11, 12–23, 24–35, 36–47, and 48–59 months), birth order (1, 2, 3, 4 and above), history of fever (occurrence of fever in the last two weeks) diarrhea in the last two weeks (an episode of diarrhea, defined as passage of three or more loose or liquid stool in 24 h [28]), childhood stunting, and wasting (stunting was defined as moderate and severe – that is below minus two standard deviations (< -2SD) from median height-for-age z-scores of reference population whilst underweight was defined as moderate and severe – that is below minus two standard deviations (< -2SD) from median weight-for-age z-score of reference population) [29]. Maternal and household characteristics include age in years (15–24, 25–34, and ≥ 35 years), educational attainment (no formal education, primary education, and secondary and higher education), and biofuel smoke exposure (dirty or clean fuel use). The use of electricity, natural gas, biogas, and kerosene for cooking were regarded as clean fuel whilst wood, straw, animal dung, and crop residues used as cooking fuels were defined as dirty fuel [30, 31]. Parity was categorized into 1, 2, 3, 4, 5 and above children, and household wealth status was categorized into poorest, poor, middle, rich and richest. The wealth index was generated through a principal component analysis that utilized data on the household’s ownership of selected assets. The household asset scores generated were then categorized into quintiles following standardization [32].

#### Community-level factors

We included six variables. One variable indicated an area of residence (i.e., urban or rural). Six continuous variables assessed community maternal anemia, community total children ever born, community wealth, community female education, community distance to a health facility and community water supply. The primary sample unit (PSU) in the DHS data was used to define a community. Individual-level data were aggregated to create continuous community-level factors [33]. The community wealth was defined as a percentage of households categorized as 60% and above of wealth index, similarly community female education was defined as the percentage of women with primary school education and above. Community maternal anemia was defined as the percentage of women with Hb levels less than 12 g/dL. Community parity was the percent of women with a fertility rate of 5 children and above and community distance to the health facility was defined as a percentage of household who perceived the distance to the nearest health facility as a big problem. Community water supply was defined as the percent of household with access to clean and safe drinking water sources specified by WHO/UNICEF [34]. All community-level variables were categorized as “low”, “medium” and “high” depending upon each variable’s tertiles.

### Statistical analyses

**Fig. 1:**
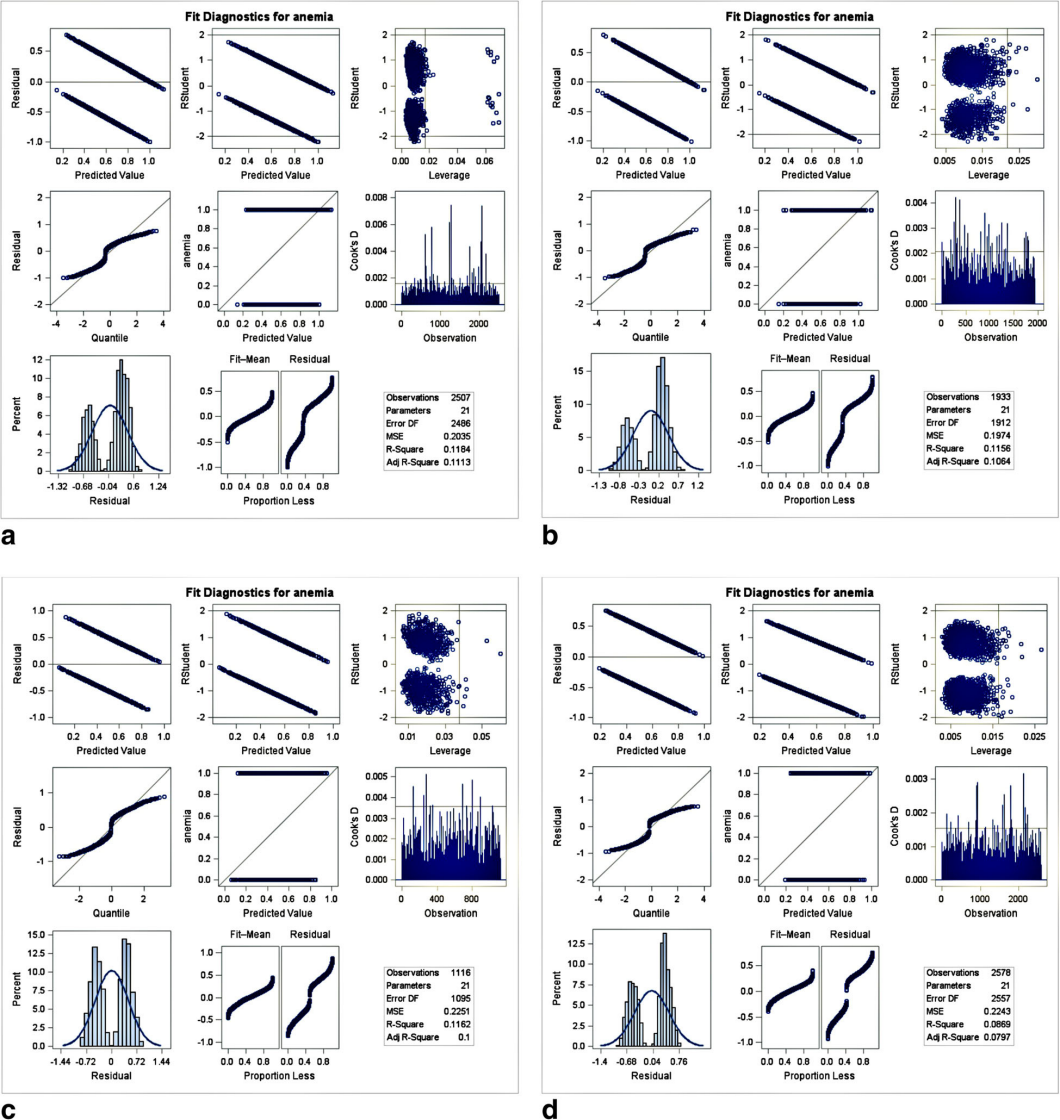
Fit Diagnostics for Childhood Anemia in (**a**) Malawi, (**b**) Mozambique, (**c**) Namibia, and (**d**) Zimbabwe

SAS software version 9.4 (SAS Institute Inc., Cary, NC, USA) was used for all statistical analysis. Due to the possible effect of clustering, data on baseline sociodemographics were weighted. Frequencies and percentages were reported for categorical variables. The differences in maternal anemia, sociodemographic, nutrition, morbidity outcomes, and community factors according to anemia status were compared using chi-square tests. Cochran-Armitage Trend Test was used to assess trends. A two-level multilevel multivariable logistic regression (MMLR) analysis with a logit-link function and binomial distribution was applied, fitting four different generalized linear mixed models. Since children from the same community may present similar characteristics than individuals from different communities, we adjusted for the correlated individual responses nested under a single community using multilevel models. To avoid large type II errors, only variables significant at *p* ≤ 0.25 in the bivariate analysis were included in this analysis [35]. Four models were constructed in this analysis. Model 1 was a null model for assessing the total variance between communities. Model 2 contained maternal anemia and individual-level factors, and model 3 contained only community-level factors. Model 4 included maternal anemia status, individual- and community-level factors. Only the final model is presented in this paper. Results of the multivariable analyses are reported in terms of adjusted odds ratios (aORs) with their *p*-values and 95% confidence intervals (CIs). Intra-class correlations (ICCs) and percentage change in variation (PCV) were reported to assess the extent to which community variances were explained in each model. Model fits were assessed using a deviation information criterion (DIC). Prior to multivariate analysis, multicollinearity was examined using variance inflation factor (VIF) (Additional file 1). Two variables (Child’s birth order and Total children ever born) had high VIF values. Figure 1 shows the fit diagnostics for childhood anemia, which showed that a lot of variation in the model was unexplained (proportion less).

### Ethics statement

The study protocols were reviewed and approved by the Malawi Health Science Research Committee, National Bioethics Commission of Mozambique (Comissao de Bioetica Nacional), Namibia Ministry of Health and Social Services, Medical Research Council of Zimbabwe and the Institutional Review Board (IRB) of ICF Macro, and the Centers for Disease Control (CDC) in Atlanta. Prior to the interview and blood sample collection, all participants provided written informed consent. Permission was granted by the DHS program for the use of the data.

## Results

### Sample characteristics

The prevalence of childhood anemia was 63.8, 70.0, 49.4, and 58.6% in Malawi, Mozambique, Namibia, and Zimbabwe respectively. At the individual- level, maternal anemia was estimated at 26.7, 53.4, 19.8 and 25.1% with community-level at 20.7, 58.6, 15.8 and 24.3% in Malawi, Mozambique, Namibia, and Zimbabwe respectively **(**Table 1**)**.

**Table 1:** Sample characteristics of childhood anemia for the four selected Southern African countries

Characteristic	Malawi	Mozambique	Namibia	Zimbabwe
	*N* = 2507	*N* = 1933	*N* = 1116	*N* = 2578
	n* (%)**	n* (%)**	n* (%)**	n* (%)**
Childhood anemia (%)	63.8	70.0	49.4	58.6
Exposure variable				
Maternal anemic status				
Nonanemic	1850 (73.3)	934 (46.6)	908 (80.2)	1898 (74.9)
Anemic	657 (26.7)	999 (53.4)	208 (19.8)	680 (25.1)
Individual-level				
Biofuel smoke exposure^**β**^				
No	17 (0.8)	91 (2.9)	352 (34.3)	552 (21.0)
Yes	2490 (99.2)	1842 (97.1)	764 (65.7)	2026 (79.0)
Sex of child				
Male	1253 (48.9)	929 (48.5)	533 (47.3)	1299 (49.9)
Female	1254 (51.1)	1004 (51.5)	583 (52.7)	1279 (50.1)
Child age in months				
6–11	268 (11.4)	247 (13.2)	153 (14.3)	394 (14.8)
12–23	616 (25.1)	502 (26.0)	294 (25.1)	628 (23.9)
24–35	579 (22.2)	459 (23.3)	253 (22.4)	627 (24.8)
36–47	586 (23.0)	377 (19.9)	234 (21.0)	509 (20.5)
48–59	458 (18.3)	348 (17.6)	182 (17.2)	420 (16.0)
Child’s birth order				
1	498 (20.8)	472 (23.8)	351 (32.6)	832(32.1)
2	437 (17.8)	347 (17.3)	278 (25.2)	680 (26.1)
3	405 (16.2)	284 (14.2)	177 (16.1)	449 (17.9)
4+	1167 (45.2)	830 (44.7)	310 (26.1)	617 (23.9)
Recent episodes of fever				
No	1590 (62.5)	1658 (84.7)	310 (70.5)	2285 (88.4)
Yes	917 (37.5)	275 (15.3)	329 (29.5)	293 (11.6)
Recent episodes of diarrhea				
No	2084 (83.5)	1709 (87.3)	868 (76.5)	2158 (83.0)
Yes	423 (16.5)	224 (12.7)	248 (23.5)	420 (17.0)
Children are stunted				
No	1303 (51.2)	1062 (50.7)	855 (77.2)	1690 (65.5)
Yes	1204 (48.8)	871 (49.3)	261 (22.8)	888 (34.5)
Children are underweight				
No	2179 (86.8)	1657 (84.0)	976 (87.2)	2304 (89.9)
Yes	328 (13.2)	276 (16.0)	140 (12.8)	274 (10.1)
Mother’s age in years				
15–24	773 (31.1)	630 (32.8)	337 (28.0)	870 (33.8)
25–34	1182 (46.6)	817 (41.0)	477(44.7)	1227 (46.9)
35–49	552 (22.3)	486 (26.2)	302 (27.3)	481 (19.3)
Mother’s educational level				
No education	423 (17.6)	740 (40.2)	86 (5.9)	60 (2.3)
Primary	1778 (70.5)	993 (52.4)	261 (22.8)	848 (32.1)
Secondary and above	306 (11.9)	200 (7.4)	769 (71.3)	1670 (65.6)
Total children ever born				
1	361 (15.0)	374 (19.1)	312 (29.2)	703 (27.0)
2	451 (18.6)	348 (17.5)	286 (26.1)	723 (28.0)
3	426 (17.6)	305 (14.8)	190 (17.0)	486 (19.4)
4	403 (14.6)	262 (13.8)	115 (9.7)	308 (11.7)
5+	866 (34.2)	644 (34.8)	213 (18.0)	358 (13.9)
Household wealth index				
Poorest	484 (18.7)	431 (28.0)	247 (23.7)	676 (24.4)
Poor	623 (25.8)	445 (25.3)	244 (21.7)	562 (22.3)
Middle	610 (23.9)	406 (20.5)	235 (19.2)	501 (20.4)
Richer	509 (19.3)	415 (18.5)	248 (21.5)	514 (20.2)
Richest	281 (12.5)	236 (7.7)	142 (13.9)	325 (12.7)
Community-level (clusters)				
Place of residence				
Urban	159 (8.2)	407 (17.8)	480 (44.5)	633 (25.1)
Rural	2348 (91.8)	1526 (82.2)	636 (55.5)	1945 (74.9)
Community maternal anemia^†^				
Low anemia	2020 (79.3)	846 (41.4)	967 (884.2)	2010 (75.7)
High anemia	487 (20.7)	1087 (58.6)	149 (15.8)	568 (24.3)
Community parity^∫^				
Low	913 (38.7)	711 (30.8)	860 (79.1)	1171 (45.3)
High	1594 (61.3)	1222 (69.2)	256 (20.9)	1407 (52.7)
Community wealth^††^				
Low	2041 (79.7)	1328 (76.7)	794 (69.8)	1753 (67.8)
High	466 (20.3)	605 (23.3)	322 (30.1)	825 (32.2)
Community female education^§^				
Low	585 (24.4)	1230 (66.5)	127 (7.8)	594 (22.8)
High	1922 (75.6)	703 (33.5)	989 (92.2)	1984 (77.2)
Community distance to HF^¥^				
Low	686 (30.1)	672 (28.7)	682 (62.9)	1031 (41.0)
High	1821 (69.9)	1261 (71.3)	434 (37.1)	1547 (59.0)
Community safe water access^‡^				
Low	619 (26.3)	1336 (75.6)	434 (19.4)	497 (19.3)
High	1888 (73.7)	597 (24.4)	922 (80.6)	2081 (80.7)

Note: *unweighted frequency; **weighted percent; ^**β**^wood, straw, animal dung, and crop residues used as cooking fuels; ^**†**^ percent of women with Hb levels less than 12 g/dL; ^**∫**^ percent of women with fertility rate of 5 children and above; ^**††**^ percent of households categorized above 60% of wealth index; ^**§**^ percent of women with primary school education and above; ^**¥**^ percent of households perceived distance to the nearest health facility as a big problem; ^**‡**^ percent of household with access to clean and safe drinking water sources specified by WHO/Unicef [34]; HF, health facility

Table 2 presents results of the bivariate analysis for all four countries. The prevalence of childhood anemia was found to be significantly higher among children whose mothers were anemic, children who were exposed to biofuel smoke (Mozambique), males (Namibia), children aged 6–23 months, children with a recent history of fever (Malawi, Mozambique and Namibia), diarrhea and chronic undernutrition (Malawi, Mozambique and Zimbabwe). Furthermore, childhood anemia was also significantly higher in children whose mothers were aged 15–24 years (Malawi, Namibia and Zimbabwe), children born to mothers with no formal education (Malawi and Mozambique) and in children resident in poor and poorest households (Malawi, Mozambique and Namibia). Additionally, the prevalence was also significantly higher in children residing in rural areas (Malawi and Mozambique), communities with a higher percentage of anemic women, low parity (Malawi and Mozambique) low wealth (Malawi, Mozambique and Namibia), low female education (Malawi and Mozambique), high perceived distance to the health facility as a big problem (Malawi and Mozambique), and low access to safe water (Malawi and Mozambique).

**Table 2:** Bivariate analysis of childhood anemia for the four selected Southern African countries

Characteristic	Malawi		Mozambique		Namibia		Zimbabwe	
	Anemia*		Anemia*		Anemia*		Anemia*	
	*No %*	*Yes %*	*No %*	*Yes %*	*No %*	*Yes %*	*No %*	*Yes %*
Exposure variable								
Maternal anemic status								
No anemic	38.76	61.24^**a**^	40.04	59.96^**a**^	50.88	49.12^**b**^	44.26	55.74^**a**^
Anemic	26.18	73.82	26.23	73.77	39.42	60.58	35.74	64.26
Individual-level								
Biofuel smoke exposure^**β**^								
No	58.82	41.18	53.85	46.15^**a**^	52.56	47.44	43.12	56.88
Yes	35.30	64.70	31.87	68.13	46.99	53.01	41.71	58.29
Sex of child								
Male	34.24	65.76	32.72	67.28	45.22	54.78^**c**^	41.57	58.43
Female	36.68	63.32	33.07	66.93	51.97	48.03	42.46	57.54
Child age in months								
6–11	9.33	90.67^a⁑^	19.43	80.57^a⁑^	33.99	66.01^a⁑^	28.17	71.83^**a**⁑^
12–23	25.65	74.35	25.70	74.30	31.97	68.03	27.07	72.93
24–35	35.75	64.25	33.55	66.45	49.80	50.20	43.06	56.94
36–47	45.39	54.61	37.67	62.33	62.82	37.18	54.03	45.97
48–59	50.87	49.13	46.84	53.16	68.68	31.32	61.19	38.81
Child’s birth order								
1	31.93	68.07	32.84	67.16	44.73	55.27	41.11	58.89
2	38.52	62.01	35.45	64.55	50.36	49.64	40.88	59.12
3	38.52	61.48	31.69	68.31	51.41	48.59	45.66	54.34
4+	34.96	65.04	32.29	67.71	50.32	49.68	41.82	58.18
Recent episodes of fever								
No	39.94	60.06^**a**^	34.74	65.26^**a**^	51.59	48.41^**b**^	42.36	57.64
Yes	27.70	72.30	21.82	78.18	41.95	58.05	39.25	60.75
Recent episodes of diarrhea								
No	37.38	62.62^**a**^	33.94	66.06^**b**^	52.19	47.81^**a**^	43.28	56.72^**b**^
Yes	26.00	74.00	25.00	75.00	36.69	63.31	35.48	64.52
Children are stunted								
No	38.60	61.40^**a**^	38.14	61.86^**a**^	50.06	49.94	43.55	56.45^**c**^
Yes	32.06	67.94	26.52	73.48	44.44	55.56	39.08	60.92
Children are underweight								
No	36.71	63.29^**a**^	34.58	65.42^**a**^	48.16	51.84	42.58	57.42
Yes	27.13	72.87	22.83	77.17	52.86	47.14	37.23	62.77
Mother’s age in years								
15–24	31.44	68.56^**c**^	29.37	70.63	38.58	61.42^**a**^	36.78	63.22^**a**^
25–34	37.99	62.01	34.88	65.12	51.57	48.43	43.77	56.23
35–49	35.69	64.31	34.16	65.84	55.63	44.37	46.99	53.01
Mother’s educational level								
No education	29.79	70.21^**b**^	30.27	69.73^**a**^	47.67	52.33	38.33	61.67
Primary	35.55	64.45	31.32	68.68	47.89	52.11	39.74	60.26
Secondary and above	42.81	57.19	50.50	49.50	49.15	50.85	43.29	56.71
Total children ever born								
1	26.87	73.13^**c⁑**^	31.28	68.72	42.63	57.37^**c⁑**^	37.41	62.59^**c⁑**^
2	37.92	62.08	36.49	63.51	50.70	49.30	43.43	56.57
3	40.14	59.86	30.16	69.84	52.11	47.89	45.06	54.94
4	33.75	66.25	35.11	64.89	49.57	50.43	40.26	59.74
5+	36.26	63.74	32.30	67.70	51.64	48.36	45.53	54.47
Household wealth index								
Poorest	28.93	71.07^**a⁑**^	24.13	75.87^**a⁑**^	50.20	49.80^**c⁑**^	41.57	58.43
Poor	33.71	66.29	28.54	71.46	42.21	57.79	40.57	59.43
Middle	33.28	66.72	33.00	67.00	46.81	53.19	43.51	56.49
Richer	39.69	60.31	36.63	63.37	48.79	51.21	40.08	59.92
Richest	47.69	52.31	50.42	49.58	60.56	39.44	46.15	53.85
Community-level (clusters)								
Place of residence								
Urban	45.91	54.09^**b**^	43.00	57.00^**a**^	50.00	50.00	40.28	59.72
Rural	34.75	65.25	30.21	69.79	47.80	52.20	42.57	57.43
Community maternal anemia^†^								
Low anemia	37.23	62.77^**b**^	39.36	60.64^**a**^	50.36	49.64^**b**^	40.75	59.25^**c**^
High anemia	28.13	71.87	27.87	72.13	38.26	61.74	46.48	53.52
Community parity^∫^								
Low	41.95	58.05^**a**^	36.85	63.15^**b**^	49.19	50.81	41.59	58.41
High	31.74	68.26	30.61	69.39	47.27	52.73	42.36	57.64
Community wealth^††^								
Low	33.56	66.44^**a**^	28.31	71.69^**a**^	46.47	53.53^**c**^	42.33	57.67
High	43.78	56.22	42.98	57.02	54.35	45.65	41.33	58.67
Community female education^§^								
Low	29.40	70.60^**a**^	29.35	70.65^**a**^	44.09	55.91	41.25	58.75
High	37.30	62.70	39.12	60.88	49.34	50.66	42.24	57.76
Community distance to HF^¥^								
Low	43.00	57.00^**a**^	39.73	60.27^**a**^	50.15	49.85	41.61	58.39
High	32.62	67.38	29.26	70.74	46.54	53.46	42.28	57.72
Community safe water access^‡^								
Low	31.66	68.34^**c**^	28.44	71.56^**a**^	50.52	49.48	40.24	59.76
High	36.71	63.29	42.88	57.12	48.37	51.63	42.43	57.57

Note: ^**a**^***P*** < 0.0001; ^**b**^***P*** < 0.001; ^**c**^***P*** < 0.05; **⁑** Cochran-Armitage Trend Test – statistical trend analysis; *Children with hemoglobin levels of less than 11 g/dL adjusted for altitude; ^**β**^ wood, straw, animal dung, and crop residues used as cooking fuels ^**†**^ percent of women with Hb levels less than 12 g/dL; ^**∫**^ percent of women with fertility rate of 5 children and above; ^**††**^ percent of households categorized above 60% of wealth index; ^**§**^ percent of women with primary school education and above; ^**¥**^ percent of households perceived distance to the nearest health facility as a big problem; ^**‡**^ percent of household with access to clean and safe drinking water sources specified by WHO/Unicef [34]

### Effect of maternal anemia and other covariates on childhood anemia

Table 3 showed the results of multivariate multilevel logistic regression analyses (Model 4 only). Compared to non-anemic mother, anemic mothers were (aOR = 1.69; 95% CI: 1.37–2.13), 29% (aOR = 1.71; 95% CI: 1.37–2.13), (aOR = 1.55; 95% CI: 1.08–2.22) and (aOR = 1.52; 95% CI: 1.25–1.84) significantly more likely to have anemic children in Malawi, Mozambique, Namibia, and Zimbabwe respectively. Additionally, compared to communities with a high percentage of anemic women, communities with a low percentage of anemic women were (aOR = 1.52; 95% CI: 1.19–1.94) more likely to have anemic children in Mozambique.

**Table 3:** Measures of association and variation between individual- and community-level factors and childhood anemia

Characteristic	Malawi	Mozambique	Namibia	Zimbabwe
	aOR 95% (CI)/*p*	aOR 95% (CI)/*p*	aOR 95% (CI)/*p*	aOR 95% (CI)/*p*
Exposure variable				
Maternal anemic status				
Nonanemic	1.00	1.00	1.00	1.00
Anemic	1.69 (1.35–2.12)^**a**^	1.71 (1.37–2.13)^**a**^	1.55 (1.08–2.22)^**c**^	1.52 (1.25–1.84)^**a**^
Maternal Hb concentration	0.64 (0.55–0.55)^**a**^	0.67 (0.58–0.78)^**a**^	0.70 (0.54–0.91)^**b**^	0.75 (0.65–0.86)^**a**^
Individual-level				
Biofuel smoke exposure^β^				
No	NA	1.00	1.00	NA
Yes	NA	0.93 (0.50–1.73)	0.92 (0.58–1.45)	NA
Sex of child				
Male	1.00	NA	1.00	NA
Female	0.93 (0.78–1.11)	NA	0.76 (0.59–0.99)^a^	NA
Child age in months				
6–11	1.00	1.00	1.00	1.00
12–23	0.28 (0.17–0.44)^**a**^	0.58 (0.39–0.88)^**a**^	1.08 (0.70–1.69)	0.99 (0.74–1.33)
24–35	0.17 (0.11–0.27)^**a**^	0.36 (0.24–0.54)^a^	0.51 (0.33–0.79)^**b**^	0.46 (0.35–0.62)^**a**^
36–47	0.12 (0.07–0.19)^**a**^	0.30 (0.20–0.46)^a^	0.31 (0.20–0.49)^**a**^	0.31 (0.23–0.42)^**a**^
48–59	0.09 (0.06–0.15)^**a**^	0.22 (0.14–0.33)^a^	0.26 (0.16–0.43)^**a**^	0.25 (0.18–0.34)^**a**^
Recent episodes of fever				
No	1.00	1.00	1.00	1.00
Yes	1.57 (1.29–1.90)^**a**^	1.68 (1.19–2.37)^**a**^	1.25 (0.93–1.68)	1.09 (0.84–1.43)
Recent episodes of diarrhea				
No	1.00	1.00	1.00	1.00
Yes	0.95 (0.73–1.24)	1.07 (0.74–1.55)	1.21 (0.86–1.70)	1.07 (0.84–1.35)
Children are stunted				
No	1.00	1.00	1.00	1.00
Yes	1.33 (1.10–1.61)^**b**^	1.49 (1.17–1.89)^**a**^	1.50 (1.05–2.15)^**c**^	1.17 (0.97–1.42)
Children are underweight				
No	1.00	1.00	1.00	1.00
Yes	1.22 (0.91–1.62)	1.19 (0.84–1.69)	0.65 (0.42–1.02)	1.26 (0.94–1.69)
Mother’s age in years				
15–24	1.00	1.00	1.00	1.00
25–34	0.92 (0.75–1.14)	0.90 (0.69–1.16)	0.70 (0.51–0.95)^**c**^	0.89 (0.74–1.08)
35–49	0.96 (0.74–1.25)	0.92 (0.68–1.24)	0.66 (0.46–0.95)^**c**^	0.83 (0.65–1.06)
Mother’s educational level				
No education	1.00	1.00	1.00	NA
Primary	0.87 (0.66–1.13)	1.09 (0.85–1.40)	0.82 (0.46–1.45)	NA
Secondary and above	0.73 (0.50–1.06)	0.66 (0.42–1.04)	0.83 (0.47–1.44)	NA
Household wealth index				
Poorest	1.00	1.00	1.00	1.00
Poor	0.88 (0.50–1.06)	0.89 (0.64–1.25)	1.53 (1.01–2.30)^c^	0.96 (0.75–1.22)
Middle	0.96 (0.73–1.27)	0.80 (0.56–1.13)	1.09 (0.72–1.66)	0.82 (0.64–1.06)
Richer	0.81 (0.60–1.08)	0.84 (0.56–1.27)	1.08 (0.62–1.88)	0.91 (0.67–1.23)
Richest	0.69 (0.48–0.99)^**c**^	0.68 (0.38–1.24)	0.74 (0.36–1.53)	0.76 (0.53–1.11)
Community-level (clusters)				
Community maternal anemia^†^				
Low anemia	1.00	1.00	1.00	1.00
High anemia	1.19 (0.93–1.54)	1.52 (1.19–1.94)^a^	1.478 (0.97–2.25)	0.778 (0.63–1.03)
Place of residence				
Urban	1.00	1.00	1.00	1.00
Rural	1.07 (0.71–1.60)	1.14 (0.80–1.63)	0.87 (0.61–1.25)	0.78 (0.58–1.03)
Community wealth^††^				
Low	1.00	1.00	1.00	NA
High	0.91 (0.69–1.19)	0.78 (0.53–1.14)	0.84 (0.53–1.34)	NA
Community female education^§^				
Low	1.00	1.00	1.00	NA
High	0.82 (0.65–1.03)	1.14 (0.83–1.55)	0.801 (0.50–1.29)	NA
Community distance to HF^¥^				
Low	1.00	1.00	1.00	NA
High	1.33 (1.07–1.65)^**b**^	0.98 (0.73–1.33)	0.99 (0.72–1.36)	NA
Community safe water access^‡^				
Low	1.00	1.00	NA	1.00
High	0.90 (0.72–1.12)	0.78 (0.57–1.07)	NA	0.91 (0.73–1.14)
Measures of variation or clustering				
Community level				
[τ(SE)]^♀^	0.193 (0.084)^**c**^	0.418 (0.120)^**a**^	0.157 (0.126)	0.086 (0.048)^**c**^
[τ(SE)]	0.056 (0.080)	0.280 (0.114)^**b**^	0.070 (0.129)	0.064 (0.050)
MOR^ð^	1.52	1.85	1.46	1.32
MOR	1.25	1.66	1.29	1.27
ICC (%) ^¶^	5.54	11.27	4.55	2.55
ICC (%)	1.68	7.84	2.09	1.90
PCV**⁑** (%)	70.94	32.98	55.31	26.07
Model fit statistics				
DIC (−2log likelihood)	2935.72	2220.25	1397.00	3282.89

Notes: Note: ^**a**^***P*** < 0.0001; ^**b**^***P*** < 0.001; ^**c**^***P*** < 0.05; **‡** aOR, adjusted odds ratio; CI, confidence interval; [τ (SE)], community-level variance; SE, standard error; MOR, median odds ration; ICC, intraclass correlation; PCV, proportional change in variance; **⁑**the proportional change in variance expresses the change in the community level variance between the null model and the individual level model, and between the individual level model, and the model further including the community level covariate; DIC, deviation information criterion. Model I: unconditional model with random intercepts and had no predictors. Model II: contained a random-intercept fixed-slope and adjusted for individual-level factors. Model III: contained a random-intercept fixed-slope and adjusted for community contextual factors. Model IV: contained a random-intercept fixed-slope and controlled for both individual and community-level factors. The Bold texts indicate a statistically significant association at a *p*-value less than 0.05. ^**∫**^ percent of women with fertility rate of 5 children and above; ^**††**^ percent of households categorized above 60% of wealth index; ^**§**^ percent of women with primary school education and above; ^**¥**^ percent of households perceived distance to the nearest health facility as a big problem; ^**‡**^ percent of household with access to clean and safe drinking water sources specified by WHO/Unicef [34]; HF, health facility. ^ð^Median Odds Ratio for null Model; ¶Interclass Correlation for null Model; ^♀^community-level variance for null Model; NA variable had *p*-value greater than 0.25 at chi-square

For individual-level factors, across all countries, the odds of childhood anemia was decreased with increased age of children. In Namibia female children were significantly less likely to be anemic compared to their male counterparts (OR = 0.76; 95% CI: 0.59–0.99). Compared to children with no history of fever in the last two weeks, children with fever were (aOR = 1.57; 95% CI: 1.29–1.90 and aOR = 1.68; 95% CI: 1.19–2.37) more likely to be anemic in Malawi and Mozambique respectively. Children with no history of stunting were 19% (aOR = 0.81; 95% CI: 0.67–0.98) and 33% (aOR = 0.67; 95% CI: 0.52–0.85) less likely to be anemic in Malawi and Mozambique respectively. The richest households were (aOR = 0.69; 95% Cl = 0.48–0.99) less likely to have anemic children compared to the poorest households in Malawi. For community-level factors, children in communities with a high percentage of mothers perceiving distance to a health facility as a big problem were (aOR = 1.33; 95% CI: 1.07–1.65) more likely to be anemic in Malawi.

The measure of variation (Model 4 only) shows that about 2, 8, 2, and 2% of the community-level variance of childhood anemia were unexplained in Malawi, Mozambique, Namibia, and Zimbabwe respectively. On the other hand, the PCVs shows that 71, 33, 55, and 26% of the variance in childhood anemia across communities in Malawi, Mozambique, Namibia, and Zimbabwe respectively were explained by, individual- and community-level. The MOR showed that the odds of childhood anemia increased by about 25, 84, 9 and 90% in Malawi, Mozambique, Namibia, and Zimbabwe respectively when a mother moved from low to the high-risk community.

## Discussion

Anemia reduces oxygen transport in the body, resulting in potentially permanent growth and developmental consequences for pre-school children [1, 3–5]. In this study of four Southern African countries, maternal anemia was highly associated with childhood anemia in all the four countries after controlling for the individual and community level factors. In addition**,** the results have also demonstrated that community-level maternal anemia exhibits a strong association with childhood anemia in Mozambique.

Individual-level factors showed that with an increase in infant’s age the risk of childhood anemia decreases in all the four countries. The presence of fever two weeks prior to data collection and history of stunting (in Malawi Mozambique and Namibia), residing in poorest households (for Malawi) appeared to increase the risk of childhood anemia. For community-level factors, communities with a high percentage of women perceiving distance to the health facility as a big problem exacerbated adverse effects of maternal anemia on childhood anemia.

Our study has added a new knowledge that maternal anemia and communities with a high percentage of anemic women have adverse effects on childhood anemia beyond the pregnancy. The possible explanation is that mothers with anemia could be residing in poor households or poor communities which are indicators of socioeconomic deprivation. Thus, mothers might have problems in purchasing and providing good nutritious food for themselves as well as their children which might result in anemia due to inadequate intake of iron and other micronutrient-rich foods [36]. This is true because, within 12 months after birth, mothers and their children share a similar sociological environment, thus, their dietary patterns and quality of life may be similar [37]. Also, mothers and their children may share similar exposure to infections such as helminthiasis, malaria and other infectious diseases that may interfere with their red blood cells production and iron stores [38]. Additionally, low levels of essential minerals such as iron, zinc and folate as well as vitamin A and B12 in the breast milk of the anemic mother could also affect the Hb level of the breastfeeding child [39].

In addition, we found out that younger children were more likely to be anemic. This result is consistent with previous findings [10, 18, 40, 41]. During the infancy-childhood growth spurt (6–12 months), children are particularly venerable to anemia due to the increase growth rate and subsequent demand for nutrients. Therefore an inadequate intake of exogenous iron during this period could lead to anemia [40]. In addition, infection resulting from the ingestion of impure foods and water [42, 43] could lead to symptoms such as diarrhea, vomiting and mouth ulcers which further affect their ability to ingest and absorb iron and other micronutrients [43, 44].

As reported in previous studies [20, 36], we found that the presence of fever in the previous two weeks and stunting were positively associated with childhood anemia. Anemia can result from infections such as malaria and helminthiasis that are associated with fever [45]. Malaria and helminthiasis have also been implicated in increase RBC destruction with accompanying decreased replacement by the bone marrow [2, 46, 47]. Moreover, anemia is exacerbated by underlying inflammation, with subsequent iron imbalance and decreased erythrocyte level [48]. Previous reports have also implicated several pro-inflammatory cytokines in chronic anemia [49].

Furthermore, stunting denotes chronic food shortage, long-term effects of low iron intake and other micronutrient deficiencies, impaired immunity which is associated with low concentrations of Hb in the socioeconomically disadvantaged household [50]. In this study, living in poor and poorest households exhibited negative effects on childhood anemia in Malawi and Namibia.

Our findings also show that community-level factors increased the adverse effects of maternal anemia on childhood anemia in the Southern African countries. Communities with a high percentage of women perceiving distance to the health facility as a big problem increased the odds of childhood anemia. Distance to the nearest health facility is broadly linked to poor child health outcomes since access to health services are low. For instance, malaria and helminth infections are most common ailments in under 5 children in Sub-Saharan Africa and other developing counties, and anemia could be a complication of these diseases if not timely and effectively treated [51, 52]. In addition, unsafe drinking water supply may increase episodes of gastrointestinal infections which in turn are greatly linked to anemia due to depletion of iron stores and hemolysis of RBCs [46].

Our study is not without limitations. The cross-sectional nature of the data could not allow us to infer causality. The analysis was limited to children whose households were selected for Hb estimation.

The HemoCue system was used for measuring Hb levels to determine anemia. Additional studies may require using other Hb indices. Finally, we cannot exclude the effects of recall and social desirability bias on self-reported data.

## Conclusion

Our study results indicated that maternal anemia was significantly associated with childhood anemia in all four countries. In addition, the individual- and community-level factors increased the adverse effects of maternal anemia on childhood anemia in Southern Africa countries. Public health policy and interventions aimed at reducing childhood anemia should focus on maternal anemia with emphasis on addressing iron and other micronutrient deficiencies, infections and health care practices of mothers with children below the age of 35 months. Distance to the nearest health facility will also need to be addressed at the community level.

## Additional file

Additional file 1: VIF and Tolerance for the four selected Southern African countries. **Table S1** shows the multicollinearity using VIF and Tolerance for the four selected Southern African countries. (DOC 72 kb)

## Abbreviations

2SD: Two standard deviations
aOR: Adjusted odds ratio
CDC: Center for Disease Control
Cl: Confidence interval
DHS: Demographic and Health survey
DIC: Deviation information criterion
GLMM: Generalized linear mixed model
Hb: Hemoglobin
Hct: Hematocrit
HIV: Human immunodeficiency virus
ICCs: Intra-class correlations
IFC: International Finance Corporation
IL: Interleukin
IRB: Institutional Review Board
MMLR: Multilevel multivariable logistic regression
PCV: Percentage change in variation
PSU: Primary sampling unit
RBC: Red blood cell
SSA: Sub-Saharan Africa
TNF: Tumor necrosis factor
UNICEF: United Nations International Children’s Education Fund
VIF: Variance inflation factor
WHO: World Health Organization
