# Supplementary material for: Maternal anemia is a potential risk factor for anemia in children aged 6–59 months in Southern Africa: a multilevel analysis
Source: BMC Public Health. 2018 May 22;18:650. doi: 10.1186/s12889-018-5568-5 (PMC5964691; doi:10.1186/s12889-018-5568-5)
Supplement: Supplementary file 1 — VIF and Tolerance for the four selected Southern African countries. Table S1 shows the multicollinearity using VIF and Tolerance for the four selected Southern African countries. (DOC 72 kb) [file 12889_2018_5568_MOESM1_ESM.doc]

| Additional File 1  Table 1. VIF and Tolerance for the four selected Southern African countries | | | | | | | | | | | | | | |
| --- | --- | --- | --- | --- | --- | --- | --- | --- | --- | --- | --- | --- | --- | --- |
|  |  |  |  | |  |  | | | | |  |  | |  |
| Characteristic | | | Malawi | |  | Mozambique | |  | Namibia | |  | Zimbabwe | |  |
|  |  |  | Tol | VIF |  | Tol | VIF |  | Tol | VIF |  | Tol | VIF |  |
| Exposure variable | | |  |  |  |  |  |  |  |  |  |  |  |  |
|  | Maternal anemic status | | 0.796 | 1.256 |  | 0.869 | 1.151 |  | 0.712 | 1.404 |  | 0.992 | 1.009 |  |
| Individual-level | | |  |  |  |  |  |  |  |  |  |  |  |  |
|  | Biofuel smoke exposure**^β^** | | 0.914 | 1.094 |  | 0.724 | 1.382 |  | 0.394 | 2.536 |  | 0.319 | 3.140 |  |
|  | Sex of child | | 0.987 | 1.013 |  | 0.985 | 1.015 |  | 0.974 | 1.026 |  | 0.982 | 1.018 |  |
|  | Child age in months | | 0.802 | 1.246 |  | 0.819 | 1.221 |  | 0.826 | 1.210 |  | 0.876 | 1.142 |  |
|  | **Child’s birth order** | | **0.093** | **10.698** |  | **0.077** | **12.942** |  | **0.065** | **15.407** |  | **0.082** | **12.177** |  |
|  | Recent episodes of fever | | 0.931 | 1.074 |  | 0.922 | 1.085 |  | 0.883 | 1.132 |  | 0.980 | 1.020 |  |
|  | Recent episodes of diarrhea | | 0.873 | 1.146 |  | 0.911 | 1.097 |  | 0.826 | 1.211 |  | 0.947 | 1.056 |  |
|  | Children are stunted | | 0.883 | 1.132 |  | 0.845 | 1.183 |  | 0.755 | 1.324 |  | 0.860 | 1.162 |  |
|  | Children are underweight | | 0.895 | 1.117 |  | 0.868 | 1.152 |  | 0.769 | 1.300 |  | 0.861 | 1.162 |  |
|  | Mother’s age in years | | 0.421 | 2.371 |  | 0.442 | 2.261 |  | 0.491 | 2.037 |  | 0.460 | 2.173 |  |
|  | Mother’s educational level | | 0.735 | 1.361 |  | 0.594 | 1.683 |  | 0.658 | 1.521 |  | 0.804 | 1.244 |  |
|  | **Total children ever born** | | **0.084** | **11.953** |  | **0.074** | **13.561** |  | **0.063** | **15.973** |  | **0.082** | **12.157** |  |
|  | Household wealth index | | 0.752 | 1.329 |  | 0.374 | 2.674 |  | 0.314 | 3.184 |  | 0.451 | 2.217 |  |
| Community-level (clusters) | | |  |  |  |  |  |  |  |  |  |  |  |  |
|  | Community maternal anemia^†^ | | 0.775 | 1.290 |  | 0.853 | 1.172 |  | 0.712 | 1.405 |  | 0.975 | 1.025 |  |
|  | Place of residence | | 0.711 | 1.407 |  | 0.618 | 1.619 |  | 0.500 | 2.001 |  | 0.343 | 2.917 |  |
|  | Community parity^∫^ | | 0.558 | 1.793 |  | 0.261 | 3.830 |  | 0.321 | 3.111 |  | 0.605 | 1.652 |  |
|  | Community wealth^††^ | | 0.732 | 1.366 |  | 0.519 | 1.926 |  | 0.636 | 1.571 |  | 0.763 | 1.310 |  |
|  | Community female education^§^ | | 0.820 | 1.220 |  | 0.678 | 1.476 |  | 0.656 | 1.524 |  | 0.707 | 1.414 |  |
|  | Community distance to HF^¥^ | | 0.749 | 1.336 |  | 0.514 | 1.947 |  | 0.518 | 1.929 |  | 0.683 | 1.465 |  |
|  | Community safe water access^‡^ | | 0.868 | 1.152 |  | 0.506 | 1.979 |  | 0.787 | 1.270 |  | 0.837 | 1.194 |  |
| **Note:** Tol, tolerance; VIF, Variance Inflation Factor; HF, health facility; ^β^wood, straw, animal dung, and crop residues used as cooking fuels; ^†^ percent of women with Hb levels less than 12 g/dL; ^∫^ percent of women with fertility rate of 5 children and above; ^††^ percent of households categorized above 60% of wealth index; ^§^ percent of women with primary school education and above; ^¥^ percent of households perceived distance to the nearest health facility as a big problem; ^‡^ percent of household with access to clean and safe drinking water sources specified by WHO/Unicef [33]; **Bolded :** indicate variables with a high VIF. | | | | | | | | | | | | | | |
